# Investigation of Moisture Dissipation of Water-Foamed Asphalt and Its Influence on the Viscosity

**DOI:** 10.3390/ma13235325

**Published:** 2020-11-24

**Authors:** Ning Li, Wei Tang, Xin Yu, He Zhan, Hui Ma, Gongying Ding, Yu Zhang

**Affiliations:** 1 College of Civil and Transportation Engineering, Hohai University, Nanjing 210098, Jiangsu, China; lining24@hhu.edu.cn (N.L.); hhu_yuxin@hhu.edu.cn (X.Y.); sdzczhanhe@163.com (H.Z.); hhu_dgy@hhu.edu.cn (G.D.); zzzyyy@hhu.edu.cn (Y.Z.); 2 Jiangsu Expressway Engineering Maintenance Technology Center, Nanjing 211106, Jiangsu, China; mahui060511113@163.com

**Keywords:** water-foamed asphalt, moisture dissipation, viscosity, foaming water content, atomic force microscopy (AFM)

## Abstract

Water-foamed asphalt is capable of improving the workability of asphalt mixture. It has been extensively used for its energy-saving and emission-reducing features. Water plays an essential part in improving the workability of water-foamed asphalt mixture. However, there is still lack in profound studies of moisture dissipation of the water-foamed asphalt over time and its influence on workability. In this study, the evolutions of residual water content and rotational viscosity of the water-foamed asphalt with time were respectively measured by the analytical balance and modified rotational viscometer (RV). The atomic force microscopy (AFM) analysis was conducted to discuss the mechanism of viscosity reduction of water-foamed asphalt. The results showed that moisture evaporation is significantly influenced by the foaming water content and ambient temperature, which results in the different stabilizing time of water-foamed asphalt. When water-foamed asphalt was stabilized, the residual water inside the asphalt was less than 0.01% relative to the asphalt mass. The AFM analysis showed that the foaming process changed the distribution of wax in the water-foamed asphalt resulting in reduction of viscosity. The viscosity reduction of asphalt is highly related to the initial foaming water content. After the foaming process, the viscosity keeps stable and is independent of moisture dissipation.

## 1. Introduction

Warm-mix asphalt (WMA) mixtures, compared to the conventional asphalt (HMA) mixtures, have gained worldwide popularity recently due to the energy-saving and emission-reducing benefits [1,2]. Water-foamed asphalt (referred to as foamed asphalt in this paper), as one type of WMA technologies, is prepared by adding cold water into heated asphalt in an expansion chamber [3]. The cold water is instantaneously heated and turns into water bubbles resulting in the expansion of asphalt within a very short time. After foaming, the viscosity of foamed asphalt decreases significantly, which allows the aggregate to be coated easily and improves the workability of asphalt mixtures [4,5]. As mentioned above, only a little water is used as a foaming agent in producing water-foamed asphalt. This is beneficial not only for environmental protection, but also for the reduction of production costs, compared to other WMA technologies using additives [3,6]. Furthermore, any asphalt, such as the Trinidad Lake asphalt, crumb rubber modified asphalt, and even epoxy asphalt, can be foamed under a suitable foaming condition [7,8,9,10].

Due to the water existence in foamed asphalt, one concern raised in some studies is about the water on the performance of foamed asphalt binders or mixtures. Guo et al. evaluated the physical, rheological, and thermal properties of foamed WMA under the environmental condition of freeze-thaw (F-T) cycles. The results showed that the thermal stability and deformation resistance at high temperature were improved after F-T cycles, and the fatigue resistance was reduced [11]. It was found in the study of Liu et al. that the rheological performances of foamed asphalt in the stable state binders were similar to the unfoamed asphalt [12,13]. Using the surface free energy method, Hu et al. found that the total surface free energy and work of the cohesion of foamed WMA reduced [14]. This implies that foamed WMA mixtures are more vulnerable to moisture damage than traditional HMA mixtures. However, the test results from the research by Ali et al. indicated that increasing the foaming water content (FWC) up to 2.6% did not seem to detrimentally affect the moisture damage and rutting resistance of foamed WMA mixtures [15].

Foamed asphalt is mainly utilized to improve the construction workability of asphalt mixtures. Therefore, there are a number of studies focusing on the workability of foamed WMA. Using a self-designed and self-fabricated mixing device for asphalt mixtures, Ali et al. compared the mixing torque of foamed asphalt and traditional asphalt mixtures [16,17]. It is believed that the foamed asphalt mixture is more workable than the traditional asphalt mixture. By superpave gyratory compactor (SGC) tests, Yin et al. proposed the maximum shear stress and coatability index to evaluate the workability and coatability of foamed WMA mixture, respectively [18,19,20]. The test results indicated that the foamed WMA with the amount of optimum foaming water had better workability and coatability than HMA. Hailesilassie also found that increasing the FWC assisted in coating aggregates [21]. Bairgi investigated the tribological characteristics of foamed asphalt by the tribological experiment with a ball-on-three-plates instrument [22,23,24]. The results showed that the foaming process reduced the frictional resistance, which is beneficial for coating during the mixing and sliding ability between aggregates during the compaction of asphalt mixtures.

Viscosity is normally used to evaluate the rheological properties of asphalt. The workability of asphalt mixture has an intimate correlation with the rotational viscosity of asphalt binders at high temperature tested by a Brookfield viscometer [25,26]. For the foamed asphalt, however, it is hard to measure the viscosity accurately by the standard RV test due to the volume variation of asphalt and the interference of water bubbles [27,28]. Jenkins evaluated the viscosity of foamed asphalt by a hand-held viscometer to characterize the workability [29]. Sri Sunarjono et al. measured the flow rate of foamed asphalt to calculate its viscosity, which was inspired by the standard tar viscometer [30]. The results showed that the viscosity of foamed asphalt became higher when the foam started to dissipate. However, it is difficult to confirm that the decrease of viscosity is due to the evaporation of water, since no attemperator was used during the whole test. The decrease of temperature could also cause the viscosity of foamed asphalt to increase [31]. Using the DSR equipment, the foamed asphalt had a lower apparent viscosity than the original asphalt under high shear rate [28].

At present, the continuous dissipation of moisture after foaming and its influence on the viscosity of foamed asphalt have not been deeply studied, although the workability of foamed WMA has been studied well. In this paper, the dissipation of moisture of foamed asphalt was investigated using an analytical balance. The viscosity of foamed asphalt was measured with the modified standard RV. Based on the results of residual water content and viscosity, the relation between the moisture dissipation and viscosity of foamed asphalt was discussed. In addition, using the atomic force microscopy (AFM), the micro-structure of foamed asphalt was obtained. Through an analysis of the AFM results, the mechanism of viscosity reduction of asphalt after foaming was discussed. 

The results showed that stabilizing the time of foamed asphalt significantly varies with temperature and FWC. Foam asphalt has a lower viscosity than the original asphalt. The viscosity reduction of asphalt binder is highly related to FWC in the foaming process, but it is independent of the residual water content of asphalt in the moisture dissipation process. This may be due to the fact that the foaming process promotes the distribution of wax molecules in asphalt and decreases the internal friction force among molecules. Based on the obtained results, this study is expected to provide guidance for using the foamed WMA technology in engineering practices.

## 2. Material Preparation and Test Methods

### 2.1. Preparation of Foamed Asphalt

An original asphalt meeting PG 64-22 specification was selected as a base material for foaming. Table 1 presents the properties of the sample.

Foamed asphalt was produced by a laboratory foaming machine (XLB10P, XCMG, Xuzhou, China). The FWC in asphalt was controlled precisely by adjusting the flow rate of base asphalt and water. Before the foaming process, the base asphalt was heated to 150 °C. Then, the foaming water contents were set at 1%, 3%, and 5% by the mass of asphalt [5,32].

### 2.2. Experimental Works

#### 2.2.1. Calculation of Water Content of Foamed Asphalt

The foamed asphalt was prepared and sprayed into a preheated jar. Subsequently, the foamed asphalt was kept under two temperatures, 120 and 140 °C. During heat preservation, the samples were weighed regularly by an analytical balance and the water loss was recorded until the weight of the sample becomes stable. The weight loss of the original asphalt under these two temperatures was also recorded to eliminate the disturbance from the asphalt evaporation. It was found that the mass of the original asphalt kept at a constant. The water content of foamed asphalt is calculated by Equation (1):

(1) $$WC_{i}=\frac{(M_{i}-m_{c})-m_{a}}{M_{i}-m_{c}}\times 100\%$$

where *WC*_i_ is the water content in foamed asphalt by weight at a certain moment (%); *M*_i_ is the total weight (g) of the jar and foamed asphalt at a corresponding moment; *m*_c_ and *m*_a_ are the weight (g) of the dried jar and the original asphalt before foaming, respectively.

#### 2.2.2. Determination of Viscosity

The rotational viscosity (RV) of asphalt has been commonly employed to assess the workability of asphalt mixture. Based on the RV test results, the mixing and compaction temperature for the asphalt mixture could be determined. However, the standard RV test is not suitable for the foamed asphalt, since the stress response of foamed asphalt during the RV test would be interfered by the irregular and unstable bubbles between the spindle and inner wall of the viscometer cup [28]. In addition, the foamed asphalt would overflow from the sample container when the spindle rotates.

**Figure 1:**
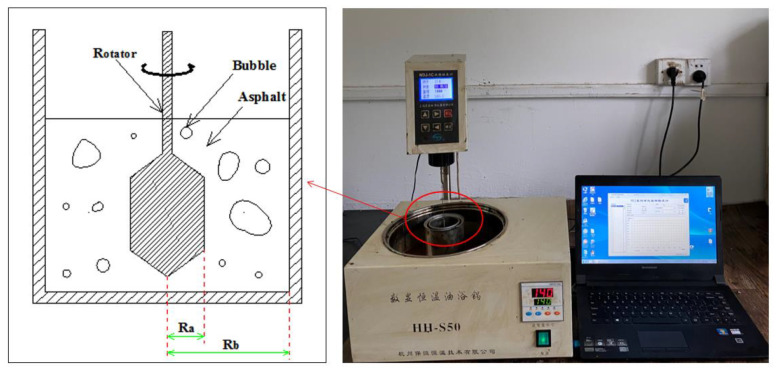
Test schematic diagram (**left**) and modified rotational viscosity (RV) test (**right**).

To solve the problems mentioned above, the RV test device was modified to assess the viscosity of foamed asphalt. Rather than the standard viscometer cup, an iron jar with 40 mm in radius (Rb = 40 mm) was adopted to enlarge the space between the spindle and inner wall. Therefore, the disturbances from the water bubbles and volume change could be reduced as much as possible. The modified RV test and its test schematic diagram are shown in Figure 1. The test data of viscosity were recorded every 9 s at 140 °C. The standard spindle (21#, Ra = 8.35 mm) was used and its rotation speed was 50 rpm. The duration time of testing was 2 h and the real-time data were recorded automatically.

#### 2.2.3. AFM Test Method

The atomic force microscope (AFM) has been commonly employed to analyze the microstructure and mechanical properties through a sharp tip probing sample in micro or nano scales [33]. In this study, the AFM analysis was carried out for the foamed asphalt to reveal the mechanism of viscosity reduction. The AFM equipment (Dimesion Edge, Bruker, Germany) was used. The probe has a silicon-coated tip with a height of 10~15 μm and a radius of 8 nm. The cantilever holding these tips had a drive frequency of 300 kHz and a spring constant of 40 N/m. For each sample, four locations were detected in the tapping mode. The obtained scanning images were processed using the NanoScope Analysis and Image-pro Plus (IPP) software.

The foamed asphalt samples with FWC of 3% were kept in a preheated oven at 140 °C for 105 and 210 min, respectively. The original asphalt sample was also tested for comparison. The AFM samples were prepared in accordance with the guidelines reported by Qtaish et al. [34,35]. Four strips of heat-resistant tapes about 20 mm in length were placed on top of a cleaned glass slide to make a square area. About 0.2 mL of the asphalt binder was injected into the square area by a syringe. Then, the glass slide was kept in an oven under 140 °C and the asphalt binder flowed to a consistent thickness over the square area. After 4~6 min, the glass slide was cooled down to an ambient temperature. After cooling, the heat-resistant tapes were removed from the glass slide.

## 3. Results and Discussion

### 3.1. Moisture Dissipation of Foamed Asphalt

**Figure 2:**
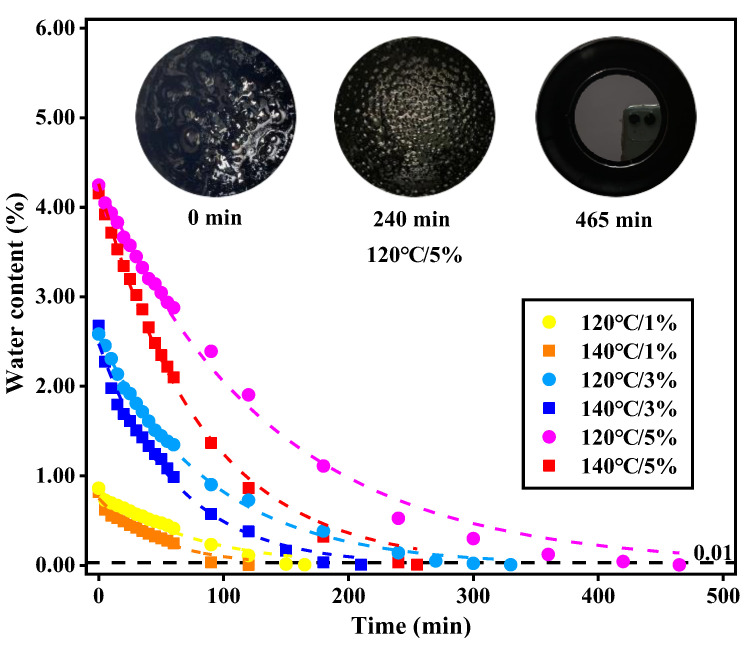
Evolutions of residual water content of the foamed asphalt over time.

The evolution of residual water content of the foamed asphalt over time is plotted in Figure 2. The label “120 °C/1%”, represents the foamed asphalt sample with FWC of 1% under the temperature of 120 °C.

From Figure 2, it can be seen that the moisture dissipated rapidly from the asphalt in the beginning. With the increase in time, the rate of moisture dissipation decreased gradually. The images shown in Figure 2 were the morphology of the foamed asphalt (120 °C/5%) after 0, 240, and 465 min. It is clear that the water bubbles with a big size were not stable and broke continuously. Then, the rest of the water bubbles were all with a small size and their rupture times became longer. At the end, there were no visible bubbles on the surface of the sample. The residual water contents were all less than 0.01% by mass. During the process of defoaming, the volume of the bubbles reduced and the bubble film appeared much thicker [36]. The bubbles owning a thicker film were more stable and could not easily exude from the asphalt binder. Therefore, the rate of moisture dissipation was slowed down [37].

Based on the data from Figure 2, the exponential relationship between the residual water content of the foamed asphalt and time is expressed by Equation (2). The fitting equations and coefficients are listed in Table 2.

*y* = *c*e^−*rt*^ (2)

where *y* is the residual water content (%) of the foamed asphalt; *t* is time (min); *c* and *r* are the fitting coefficients.

**Figure 3:**
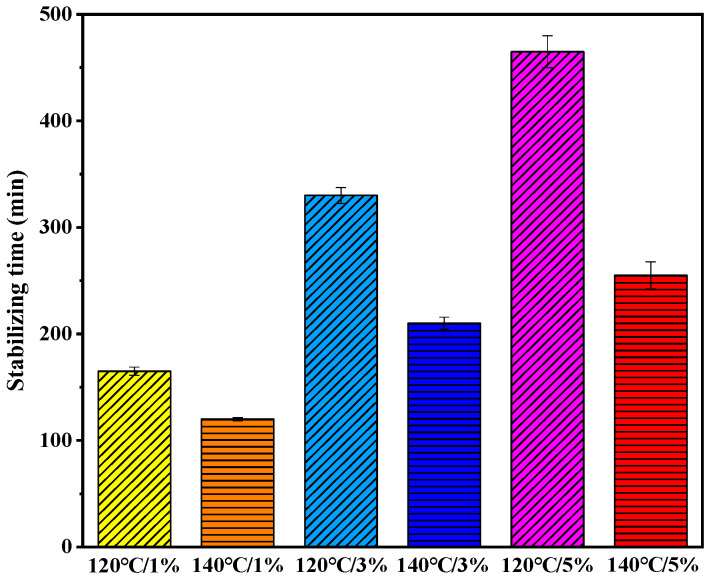
Stabilizing time of foamed asphalt under different conditions.

From Figure 2, it can be seen that the stabilizing time required by moisture dissipation varied with the FWC and temperature. Figure 3 shows the stabilizing times under different conditions.

In Figure 3, the samples with more FWC required a longer time to reach the constant weight regardless of temperature. In particular, the stabilizing time of the sample (120 °C/5%) was 465 min, which is nearly three times more than that of the sample (120 °C/1%), 165 min. The stabilizing time of the samples with FWC of 3% changed in the range from 210 to 330 min when the temperature decreased from 140 to 120 °C. The samples under higher temperature had a shorter stabilizing time. Since the viscosity of the asphalt became lower at a higher temperature, it weakened the strength of water bubbles film on the surface.

In the engineering practice, when using foaming technology to produce WMA, the residual water content of the foamed asphalt mixture may vary significantly according to the fluctuation of construction parameters, such as mixing temperature and transportation time. The obtained results are valuable to guide the construction of the foamed asphalt mixture. For instance, if the FWC is 1%, the transportation time from in-plant production to in-field paving has to be at least 120 min. Otherwise, the considerable amount of water would remain inside the asphalt mixture, resulting in moisture damage of the pavement.

### 3.2. Variation of Viscosity with Time

**Figure 4:**
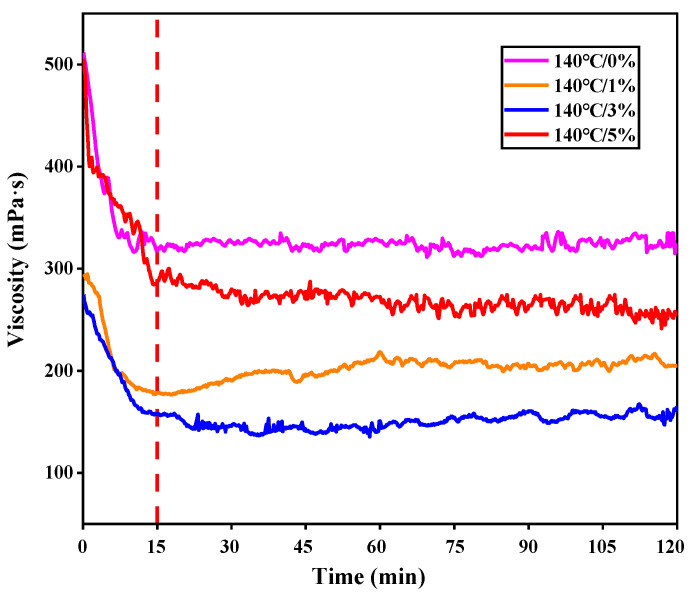
Viscosity variation of the foamed asphalt with time.

Using the modified RV test, the viscosity of foamed asphalt was determined and presented in Figure 4. It is clear that the viscosity of all samples decreased quickly within the first 15 min, since asphalt, as a viscoelastic material, needs time to reach a steady laminar flow state. After 15 min, the viscosity of foamed asphalt kept stable basically until the end of testing. In the stable state, the viscosity of the water-foamed asphalt was generally lower than that of the original asphalt, but it did not vary monotonously with the increasing FWC. It is noteworthy here that the sample with 3% FWC had the lowest viscosity, which is nearly half of the original asphalt (control sample).

**Figure 5:**
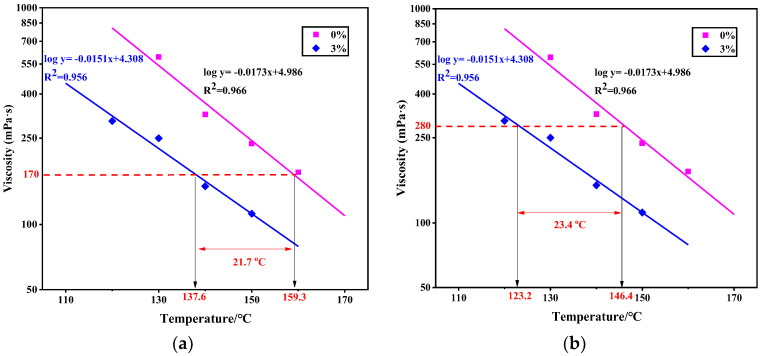
Viscosity versus temperature of the original and foamed asphalt binders: (**a**) Determination of mixing temperature; (**b**) determination of compaction temperature.

The viscosity of the original and foamed asphalt binders at various temperatures are compared in Figure 5. Based on the regression equations, the mixing and compaction temperatures could be determined, when using the viscosities of 170 and 280 mPa∙s (cP) as reference, respectively. Compared to HMA, the foamed WMA can reduce the mixing temperature of 21.7 °C and compaction temperature of 23.4 °C. It is believed that the foaming process is able to improve the workability of foamed asphalt mixture. An optimal FWC can be determined by the viscosity measured in the stable stage.

### 3.3. Relationship between Moisture Dissipation and Viscosity

**Figure 6:**
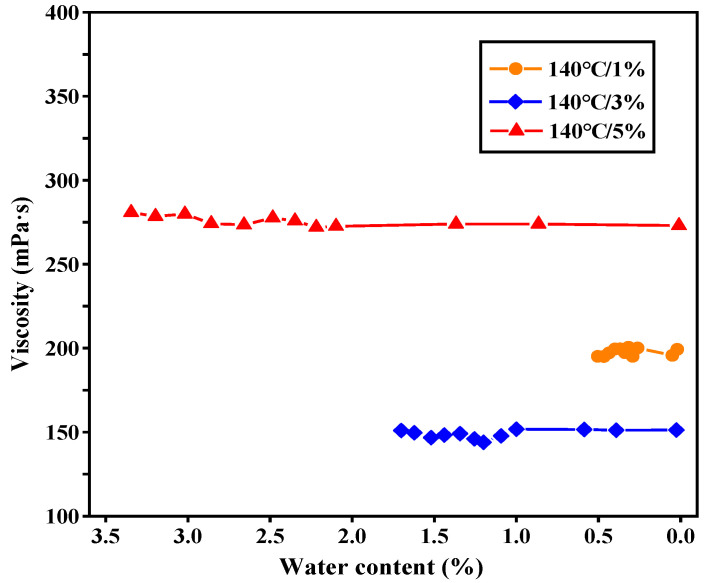
Viscosity versus water content of foamed asphalt.

When combining Figure 2 and Figure 4, the relationship between the viscosity in the stable stage and water content of foamed asphalt could be found, as presented in Figure 6. The results showed that during the process of moisture dissipation, the viscosity of foamed asphalt kept constant basically and was independent of its residual water content. It is supposed here that the viscosity reduction of foamed asphalt only depends on the FWC, and has no relevance to the moisture dissipation. This is the probable reason why after several hours of transportation, the foamed asphalt mixture still has good workability during compaction [18].

### 3.4. Micromorphology Evolution of Foamed Asphalt

**Figure 7:**
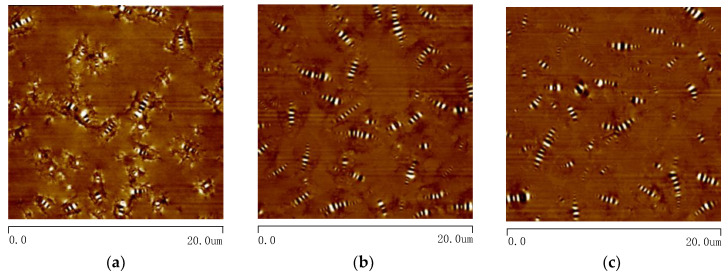
Two-dimensional atomic force microscopy (AFM) images of original and foamed asphalt binders at different moments: (**a**) 0%–0 min; (**b**) 3%–105 min; (**c**) 3%–210 min.

**Figure 8:**
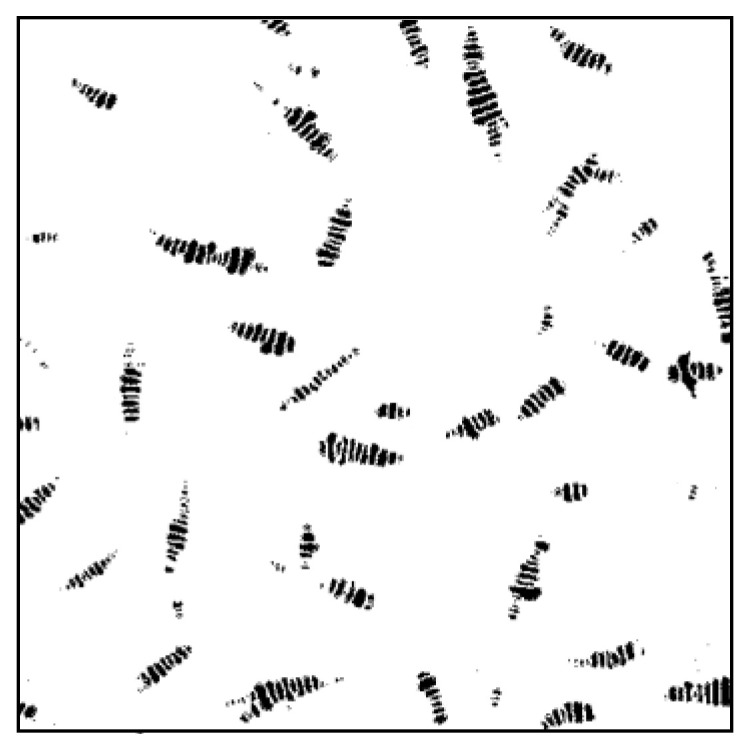
Image post-processing of the bee structure by Image-pro Plus (3%–105 min).

Two-dimensional AFM images of the asphalt samples are shown in Figure 7. The typical structures of alternating black and white stripes appeared in the images. The appearance of the structure resembles the shape of a bee, so it is called a “bee structure”. The bee structures firstly mentioned in Lober’s report were clearly observed [38]. After foaming, the number and size of the bee structure changed significantly compared to the original asphalt. Using the IPP software, the bee structures were marked out and presented in Figure 8. The total area of the bee structure to the image, as well as the number and average area of a single bee structure were calculated and listed in Table 3.

As seen from Table 3, the bee structure total area of the asphalt binder has no obvious change before and after the foaming process. However, for the foamed asphalt, a single bee structure became smaller and its distribution was more uniform, leading to the increase of the bee structure number in a certain area. With the dissipation of moisture (105 to 210 min after foaming), the number and average area of the bee structure in foamed asphalt has no significant change. The evolution of bee structure is similar to that of the viscosity in foamed asphalt. From the results of the bee structure, it can be stated that the bee structures split and distribute more widely in asphalt binder by using the foaming technology. This is an irreversible process, even though the water evaporates completely.

Many studies show that the crystalline wax, as the component of bee structure, attributes to the formation of bee structures [39,40,41]. From the variation of bee structure parameters, it can be deduced that the foaming process may promote the distribution of wax molecules inside the asphalt binder and decrease the internal friction force among molecules. This maybe the reason why the viscosity of foamed asphalt is lower compared to the original asphalt, and this would not change with moisture dissipation. In the future, more experimental works are required to explore the mechanism of the improvement of wax distribution due to the foaming process and the relationship between the bee structure parameters and viscosity of asphalt.

## 4. Conclusions

In this study, the influence of moisture dissipation from the foamed asphalt on viscosity was investigated by conducting the modified RV tests. The mechanism of viscosity reduction was explored by the results of the AFM test. The main conclusions are as follows:

- During the process of moisture dissipation, the exponential equation is proposed to describe the residual water content of foamed asphalt. The stabilizing time for foamed asphalt shows a significant variation with FWC and the preserving temperature. According to the stabilizing time, the interval time from in-plant mixing to in-field paving can be determined. After stabilizing, the residual water content of foamed asphalt is less than 0.01% by the asphalt mass.
- The modified RV is applicable to the viscosity measurement of the foamed asphalt. The viscosity reduction of asphalt binder is highly related to FWC in the foaming process, but it is independent of the residual water content of asphalt in the moisture dissipation process. This gives a reasonable explanation why the foamed asphalt mixture could maintain good workability (construction temperature reduced by more than 20 °C) throughout the whole construction process.
- Based on the AFM results, the bee structure of foamed asphalt becomes smaller and exhibits a uniform distribution compared to the original asphalt. It is clear that the foaming process promotes the distribution of wax molecules in asphalt and decreases the internal friction force among molecules, resulting in the decrease of the viscosity.

## Figures and Tables

**Table 1:** Properties of the PG 64–22 asphalt.

Properties	Test Results	Standard Specification
Penetration/0.1 mm (25 °C, 100 g, 5 s)	75	ASTM D 5
Ductility/cm (5 cm/min, 15 °C)	>100	ASTM D 113
Softening point/°C (TR&B)	48.5	ASTM D 36
Rotational viscosity (mPa·s, 135 °C)	448.1	ASTM D 4402
G*/sinδ (kPa, 64 °C)	1.76	AASHTO T315
PG grade	PG 64–22	AASHTO T315

**Table 2:** Exponential fitting results.

Samples	Fitting Equation	*c*	*r*	*R* ^2^
120 °C/1%	*y* = 0.85e^−0.0137*t*^	0.85	0.0137	0.964
140 °C/1%	*y* = 0.75e^−0.0205*t*^	0.75	0.0205	0.954
120 °C/3%	*y* = 2.63e^−0.0116*t*^	2.63	0.0116	0.998
140 °C/3%	*y* = 2.54e^−0.0165*t*^	2.54	0.0165	0.986
120 °C/5%	*y* = 4.48e^−0.0076*t*^	4.48	0.0076	0.995
140 °C/5%	*y* = 4.43e^−0.0126*t*^	4.43	0.0126	0.995

**Table 3:** Parameters of the bee structure.

Asphalt Types	Percent of Total Area/%	Number	Average Area/um^2^
0%–0 min	6.10	16	1.525
3%–105 min	6.50	38	0.684
3%–210 min	6.20	42	0.590
